# Supplementary material for: Genome Sequences of Two Pseudomonas aeruginosa Isolates with Defects in Type III Secretion System Gene Expression from a Chronic Ankle Wound Infection
Source: Microbiol Spectr. 2021 Jul 14;9(1):10.1128/spectrum.00340-21. doi: 10.1128/spectrum.00340-21 (PMC8552725; doi:10.1128/spectrum.00340-21)
Supplement: SUPPLEMENTAL FILE 4 — Supplemental material. Download SPECTRUM00340-21_Supp_4_seq8.pdf, PDF file, 2.2 MB [file spectrum00340-21_supp_4_seq8.pdf]

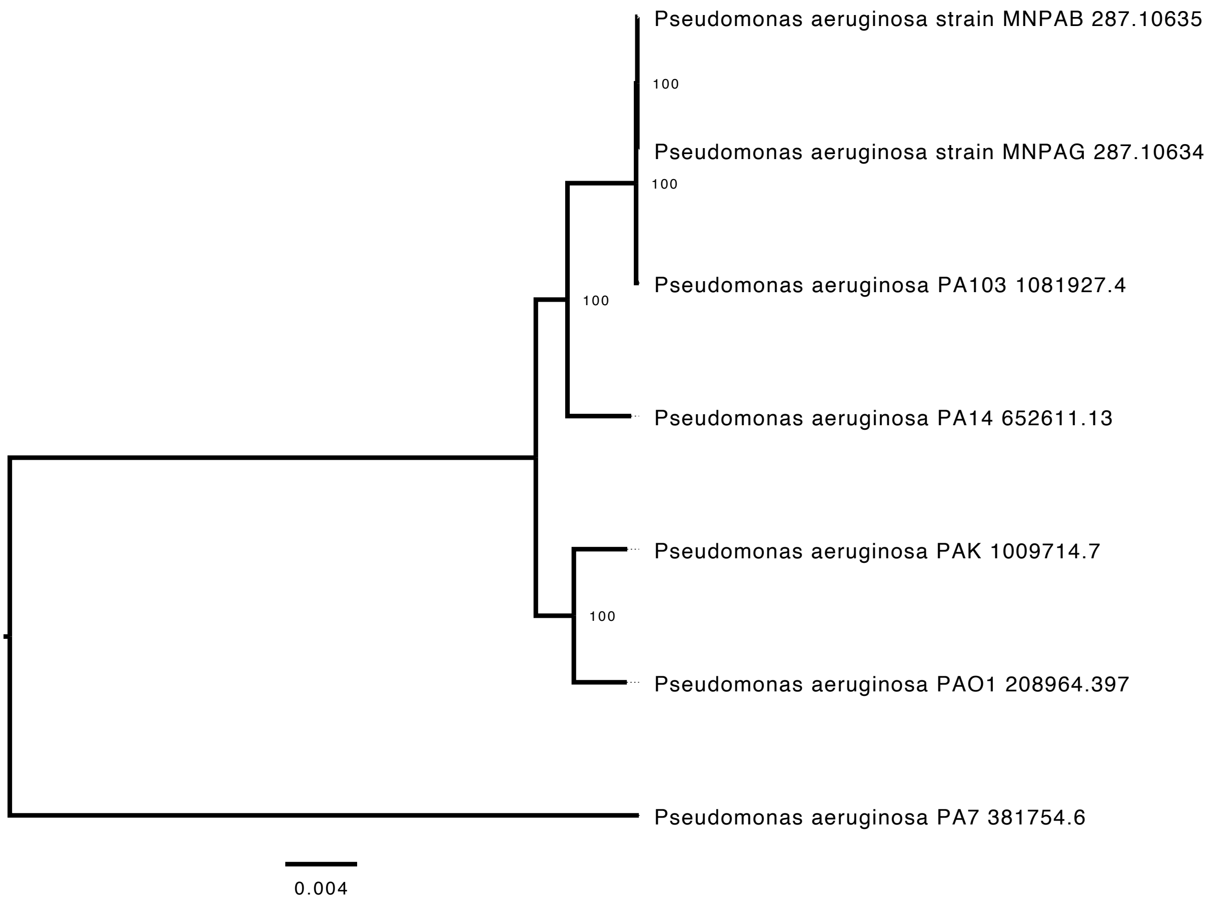

Figure S1. Phylogenetic tree of MNPAB, MNPAG, and 5 common *Pseudomonas aeruginosa* laboratory strains. The tree was built on the PATRIC platform utilizing the PGFams codon tree method, which aligns 1000 proteins and coding genes using randomized accelerated maximum likelihood (RAxML). The numbers next to branches are bootstrap values.

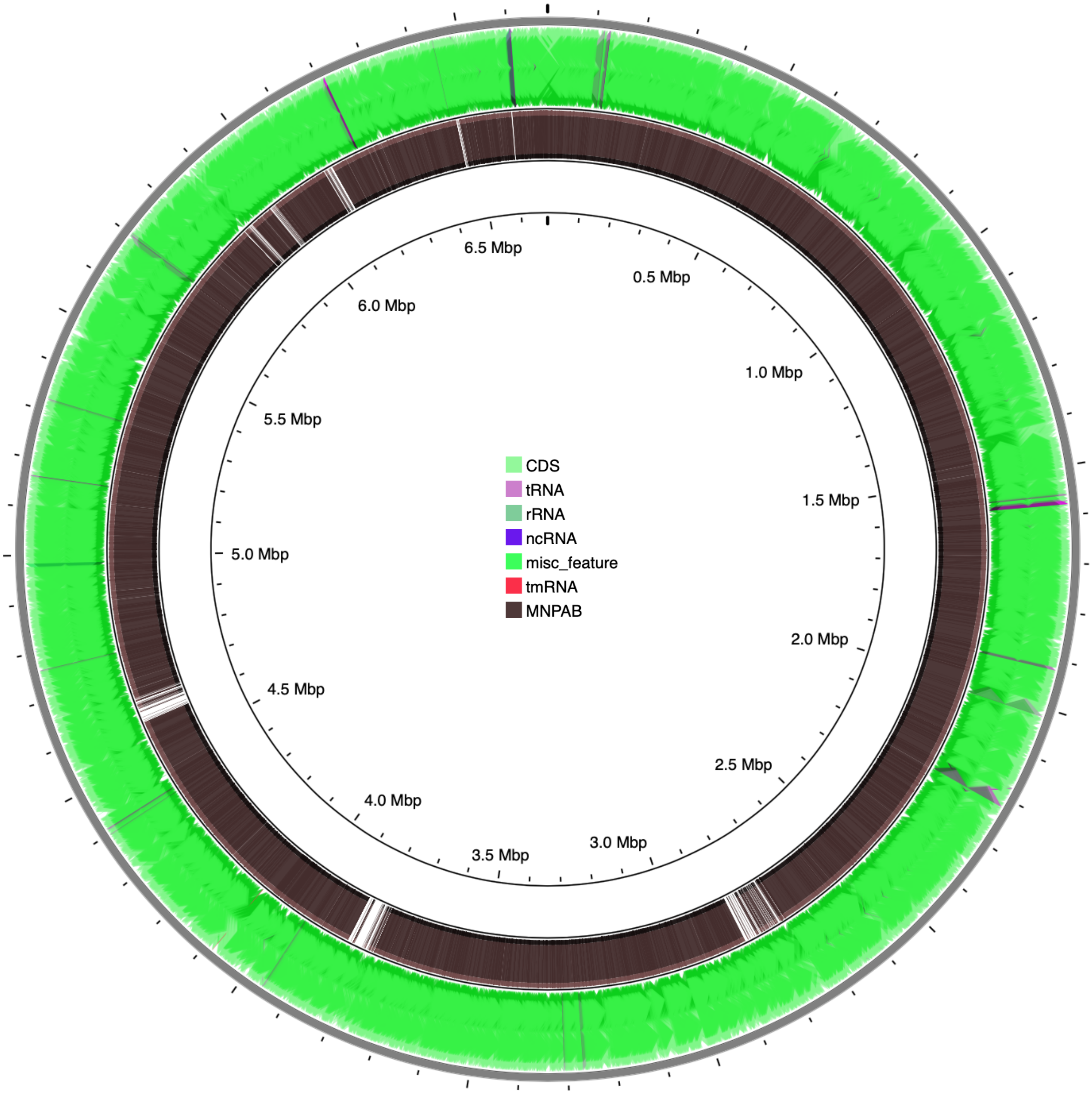

Figure S2. Circular genome map of *P. aeruginosa*. The outer green ring represents *P. aeruginosa* MNPAG and the inner brown ring represents MNPAB. The gaps in the MNPAB genome largely account for the additional 350 kb of genome sequence in MNPAG.

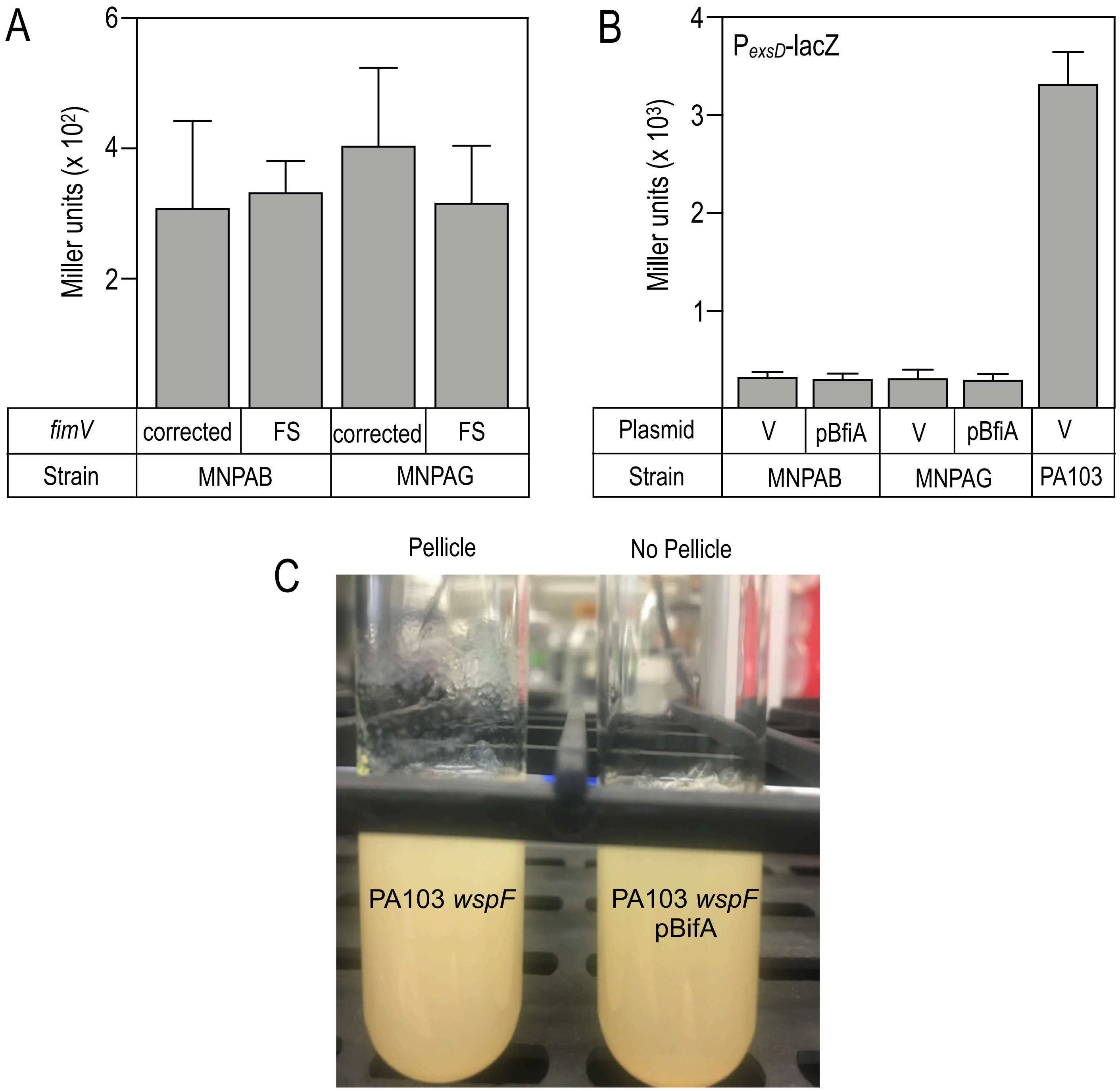

Figure S3. (A) MNPAB and MNPAG have a frameshift mutation in *fimV* (indicated as FS). The frameshift was corrected (indicated as corrected) in each background and the strains were cultured in tryptic soy broth supplemented with EGTA and assayed for ExsA-dependent reporter activity (P_{exsD}-*lacZ*). (B) MNPAB and MNPAG have a frameshift mutation in *bfiA.* Strains were transformed with either a control plasmid (V) or a BfiA expression plasmid (pBfiA) and assayed for P_{exsD}-*lacZ* reporter activity. (C) A control demonstrating that the expression of the BifA c-di-GMP phosphodiesterase reduces pellicle formation in a PA103 *wspF* mutant, which has elevated levels of c-di-GMP that promote pellicle formation.
